# PtdIns(3,4,5)P_3_-Dependent and -Independent Roles for PTEN in the Control of Cell Migration

**DOI:** 10.1016/j.cub.2006.12.026

**Published:** 2007-01-23

**Authors:** Nick R. Leslie, Xuesong Yang, C. Peter Downes, Cornelis J. Weijer

**Affiliations:** 1 Division of Molecular Physiology and University of Dundee, Dundee, DD1 5EH, Scotland, United Kingdom; 2 Division of Cell and Developmental Biology, University of Dundee, Dundee, DD1 5EH, Scotland, United Kingdom

**Keywords:** CELLBIO, SIGNALING

## Abstract

**Background:**

Phosphatase and tensin homolog (PTEN) mediates many of its effects on proliferation, growth, survival, and migration through its PtdIns(3,4,5)P_3_ lipid phosphatase activity, suppressing phosphoinositide 3-kinase (PI3K)-dependent signaling pathways. PTEN also possesses a protein phosphatase activity, the role of which is less well characterized.

**Results:**

We have investigated the role of PTEN in the control of cell migration of mesoderm cells ingressing through the primitive streak in the chick embryo. Overexpression of PTEN strongly inhibits the epithelial-to-mesenchymal transition (EMT) of mesoderm cells ingressing through the anterior and middle primitive streak, but it does not affect EMT of cells located in the posterior streak. The inhibitory activity on EMT is completely dependent on targeting PTEN through its C-terminal PDZ binding site, but can be achieved by a PTEN mutant (PTEN G129E) with only protein phosphatase activity. Expression either of PTEN lacking the PDZ binding site or of the PTEN C2 domain, or inhibition of PI3K through specific inhibitors, does not inhibit EMT, but results in a loss of both cell polarity and directional migration of mesoderm cells. The PTEN-related protein TPTE, which normally lacks any detectable lipid and protein phosphatase activity, can be reactivated through mutation, and only this reactivated mutant leads to nondirectional migration of these cells in vivo.

**Conclusions:**

PTEN modulates cell migration of mesoderm cells in the chick embryo through at least two distinct mechanisms: controlling EMT, which involves its protein phosphatase activity; and controlling the directional motility of mesoderm cells, through its lipid phosphatase activity.

## Introduction

PTEN is one of the most frequently lost tumor suppressors in human cancers and has been shown to have many diverse effects on cellular behavior, acting in many cell types to inhibit cellular survival, growth, and motility 1, 2, 3. PTEN is a lipid phosphatase that acts through metabolism of PtdIns(3,4,5)P_3_ to inhibit signaling pathways and biological processes reliant upon this second messenger. As part of the PI3K/Akt signaling pathway, PtdIns(3,4,5)P_3_ is recognized to play an important role in the regulation of many cellular processes that are antagonized by PTEN, including proliferation, growth and survival, and migration [2]. Thus, it seems clear that many of the effects of PTEN on cellular behavior are largely mediated through regulation of PtdIns(3,4,5)P_3_-dependent signaling. However, other potential mechanisms of action of PTEN have been identified, including the activity of the phosphatase against protein substrates [4] and also a phosphatase-independent capability to inhibit cell migration in a scratch-wound assay, an inhibition mediated by the C2 domain of PTEN [5]. Although the physiological significance of PtdIns(3,4,5)P_3_-independent effects of PTEN is controversial 6, 7, 8, 9, much of the evidence supporting a physiological role for the PtdIns(3,4,5)P_3_-independent activity of PTEN has come from studies of cell motility. Recent work confirmed the findings 8, 10, 11 that PTEN G129E, lacking PtdIns(3,4,5)P_3_ phosphatase activity, could inhibit cell motility as efficiently as the wild-type enzyme, when expressed through microinjection of expression constructs into PTEN null glioblastoma cells [5]. This study went on to show that this property could be seen also through expression of only the C2 domain of PTEN, and it implicated the protein phosphatase activity of PTEN in autodephosphorylation of the inhibitory phosphorylation sites in the C terminus of PTEN [5].

A role for PTEN in the control of cell polarization has been well documented during chemotaxis of *Dictyostelium* up a cAMP gradient. Deletion of PTEN results in increased and spatially extended PtdIns(3,4,5)P_3_ production at the leading edge of cells migrating in a gradient of cAMP 12, 13. This increased PtdIns(3,4,5)P_3_ domain results in defective polarization of the cells in the direction of the gradient 12, 13. In zebrafish embryos, PI3K activity has been implicated in the directional migration of invaginating mesoderm cells toward the anterior, where inhibition of PI3K results in loss of polarity and reduced migration speed [14]. In mice, deletion of PTEN results in lethality at the early stages of gastrulation before somitogenesis [15], but the detailed effects on differentiation and migration of cells have not been identified. Studies of mouse embryonic fibroblasts (MEFs) and B lymphocytes lacking the *PTEN* gene have found that these cells migrate faster than wild-type counterparts in culture, indicating a physiological role for PTEN in the suppression of cell motility 6, 16. Re-expression of PTEN in mammalian cells lacking the enzyme has been found to inhibit the motility of several lineages of such cells, including mouse embryo fibroblasts and tumor-derived cells of glial, prostate, and T cell origin 6, 8, 17, 18, although most of these studies have not addressed the mechanism of action of PTEN.

We chose to address the effect of overexpression of PTEN in mesoderm cells destined to become somites, migrating away from the primitive streak of a developing chick embryo. The migration of these cells has been shown to be controlled by chemoattractant and repellent responses to FGF4 and FGF8, respectively [19]. In the current experiments, the migration of primitive-streak cells transfected with green fluorescent protein (GFP) fusion proteins with PTEN and several PTEN mutants was followed over time by using fluorescence time-lapse microscopy, allowing a detailed characterization of the migration behavior of these cells and the demonstration that PTEN has two separable mechanism of action in this assay.

## Results

### Inhibition of Migration by PTEN

**Figure 1:**
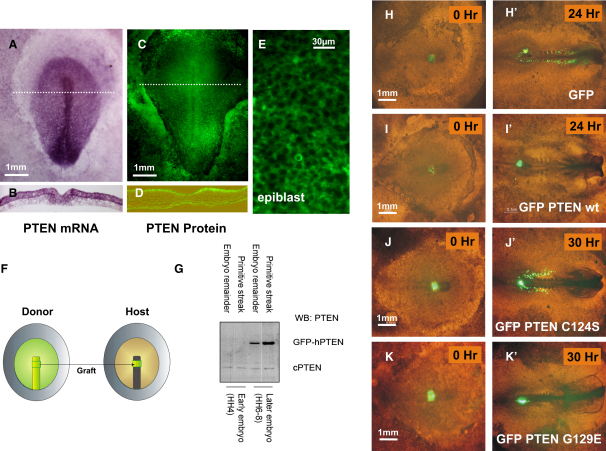
PTEN Inhibits the Outward Migration of Primitive-Streak Cells during Chick Embryogenesis (A–E) Analysis of endogenous-PTEN expression in the stage 4 (HH4) chick embryo. (A) and (B) show in situ hybridization for PTEN mRNA. (C)–(E) show immunostaining for PTEN protein. Sections of the embryos as indicated by a dashed white line in (A) and (C) are shown in (B) and (D). (F) Cartoon of the chick-embryo migration assay. Stage 4 (HH4) Chick embryos are transfected with expression vectors encoding the green fluorescent protein (GFP) or a GFP fusion protein. Transfected primitive-streak pieces (in this case, anterior streak) were grafted to replace primitive-streak tissue in an untransfected host embryo, after which development was observed by bright-field and fluorescence time-lapse microscopy. (G) The overexpression of GFP-PTEN in transfected embryos is shown by western blotting for PTEN with the pooled protein from four dissected primitive-streak fragments, immediately before and 18 hr after transfection. (H–K) Migration of anterior-streak cells expressing GFP (H), wild-type GFP-PTEN fusion protein (I), GFP-PTEN C124S (phosphatase dead) (J), and GFP-PTEN G129E (lipid phosphatase activity dead, protein phosphatase activity retained) (K) was observed over 24 or 30 hr as indicated. Initial (t = 0) and final (t = 24 hr or 30 hr) images are shown of the migration assay merging bright-field and fluorescent images, allowing the outward migration of green fluorescently marked cells to be observed. Note that in the GFP control and GFP PTEN C124S experiments, transfected anterior-streak cells have moved out of the streak and aligned themselves on both sides of the embryo's midline in the forming somites. The cells expressing the GFP-PTEN wt or G129E constructs have failed to migrate out of the streak.

We addressed the effects of phosphatase and tensin homolog (PTEN) expression upon the outward migration of cells from the anterior primitive streak during chick-embryo development (Figure 1 and Movie S1 in the Supplemental Data available online). In this assay, an embryo is transfected by electroporation and a graft of transfected cells from the primitive streak is made into an untransfected host embryo before the outward migration of these labeled cells is observed by timelapse fluorescence microscopy. In these experiments, overexpression of either PTEN or a GFP-PTEN fusion protein caused a dramatic inhibition of the migration of transfected anterior primitive-streak cells away from the primitive streak, contrasting with cells transfected with GFP alone. Anterior-streak cells transfected with GFP alone show a typical initial outward migration of the cells away from the streak, followed by a phase of migration back toward the midline after the regression process starts, as described before (Figure 1H, Figure S1A, and [19]). Anterior-streak cells overexpressing PTEN do not move out of the graft (Figure1I and Figure S1B).

Analysis of the expression of endogenous PTEN mRNA and protein by in situ hybridization and immunofluorescence, respectively, was performed in the developing chick embryo (Figure 1 and Figure S2). This showed very low expression levels early in development, but increasing levels during Hamburger and Hamilton (HH) stages 3–8, especially in the epiblast and primitive streak. Interestingly, PTEN protein appeared to be localized close to the apical membrane in epiblast cell sections, but at the cell periphery when observed from above, consistent with an enrichment at adherens junctions (see Figures 1D and 1E) 20, 21. Western-blot analysis of transfected embryos shows that the PTEN transgene is expressed very strongly (Figure 1G).

### PtdIns(3,4,5)P_3_ Phosphatase Activity Is Not Required for the Inhibition of Cell Migration out of the Streak by PTEN

The reliance of the PTEN effects upon phosphatase activity was investigated by expression of the active-site mutants PTEN C124S, which lacks all detectable phosphatase activity, and PTEN G129E, which has dramatically impaired lipid phosphatase activity but retains full protein phosphatase activity [22]. These experiments showed that PTEN C124S did not inhibit cell migration out of the streak and that the migration patterns of the cells that moved out were normal (Figure 1J and Figure S6A). Contrary to this, expression of PTEN G129E inhibited migration as efficiently as the wild-type protein. Transfected cells failed to migrate out of the streak (Figure 1K and Figure S6B), implying that the lipid phosphatase activity is not required for the inhibition of cell migration out of the streak.

**Figure 2:**
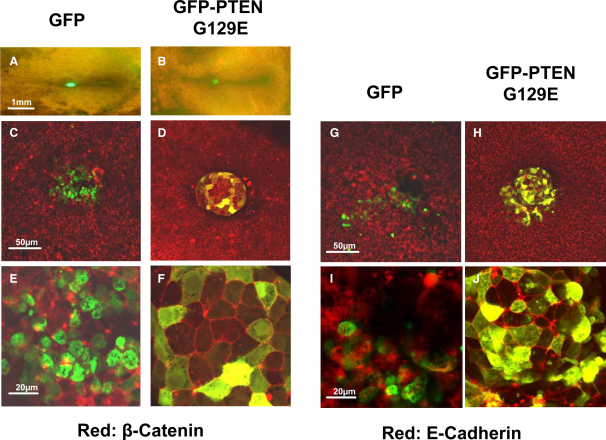
Expression of GFP-PTEN G129E Blocks EMT Cells of the primitive streak were transfected with vectors encoding either GFP (A, C, E, G, and I) or GFP-PTEN G129E (B, D, F, H, and J) before grafting into an untransfected host embryo. Development was allowed to proceed for 15 hr before analysis. (A and B) Overview images showing migration of the cells away from the streak when expressing GFP (A) and no migration when the cell express GFP-PTEN G129E (B). (C–J) High-magnification images of the grafted cells expressing GFP (C, E, G, and I) or GFP-PTEN G129E (D, F, H, and J). Transfected cells are green, and the expression— detected by antibody staining—of endogenous β-Catenin (C–F) and E-cadherin (G–J) is shown in red. The scale bars represent 1 mm in (A), 50 μm in (C) and (G), and 20 μm in (E) and (I). Expression of wild-type PTEN had the same effect in these experiments as PTEN G129E.

**Figure 3:**
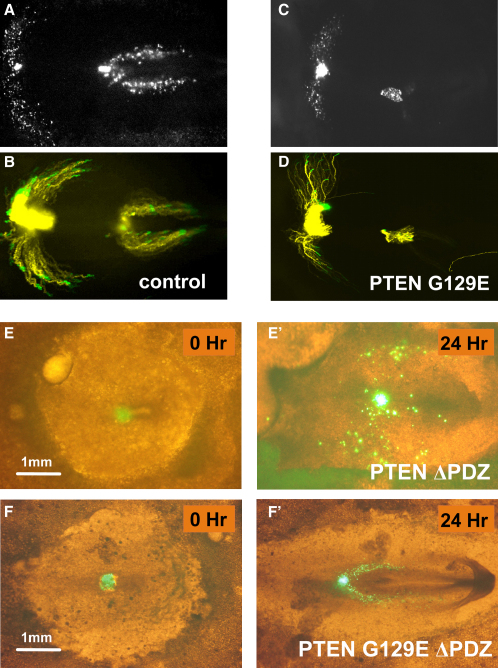
The Migration Inhibition Caused by PTEN Is Specific to Cells of the Anterior Primitive Streak and Requires Targeting Cells grafted in the anterior streak (right) and posterior streak (left) migrate when expressing GFP (A and B), but only cells in the posterior streak migrate away from the streak when expressing PTEN G129E, whereas the migration of anterior-streak cells away from the streak is inhibited (C and D). (A and C) show fluorescence images taken 15 hr after grafting. (B and D) show cell-track images depicting the migration paths of the cells during the 15 hr of the experiment. (E and F) show migration of anterior-streak cells expressing GFP-PTEN ΔPDZ (E) or GFP-PTEN G129E ΔPDZ (F) as indicated. It can be seen that in both cases, cells can migrate away from the streak, but that the migration of the cells expressing GFP-PTEN ΔPDZ is less directional (E′) than that of cells expressing GFP-PTEN G129E ΔPDZ (**F′**). Initial (t = 0) and final (t = 24 hr) images of the cell migration assay are shown; merging bright-field and fluorescent images allows the migration of green fluorescent cells to be observed in the context of the embryo.

Cells of the developing primitive streak undergo an epithelial-to-mesenchymal transition (EMT) before migrating away from the streak (23, 24 and Figure 2). In contrast, cells expressing either wild-type PTEN or PTEN G129E did not undergo an EMT. The cells appeared to be highly adhesive, did not integrate properly into the streak, stayed strongly compacted, and did not downregulate E-cadherin or β-catenin, as judged by retained immunoreactivity for these molecules at the cell periphery, relative to cells expressing GFP (Figure 2 and data not shown). As a result, the cells were unable to migrate away from the streak (Figure 1, Figure 2 and Figures S1 and S6). Interestingly, these experiments suggested that the EMT of both transfected and adjacent untransfected cells in the grafted tissue might be suppressed, indicating that secondary non-cell-autonomous effects on EMT may exist in this circumstance (Figures 2D, 2F, 2H, and 2J). Surprisingly, further experiments showed that, although the expression of wild-type or G129E PTEN strongly inhibited the migration of cells away from the anterior and middle streak, it had a much less pronounced effect on the migration of posterior cells, which were perfectly able to migrate out of the streak (Figures 3A–3D and data not shown). In the case of the G129E mutant, these posterior cells migrated relatively normally to the periphery of the embryo, suggesting that the protein phosphatase activity does not inhibit cell migration. Expression of wild-type PTEN in posterior-streak cells did not block the escape of cells from the streak, but resulted in aberrant directional migration, suggesting that the lipid phosphatase activity of PTEN is required to perturb directional migration of posterior-streak cells (see Movie S1).

In order to address the function of the endogenous PTEN protein in this context, RNA-based knockdown of PTEN expression was performed. When transfected into one half of an embryo, this was found to reduce endogenous PTEN RNA and protein levels and increase phosphorylation of the downstream kinase Akt/PKB after approximately 20 hr compared to the untransfected half of the embryo (Figures S3 and S4). PTEN siRNA transfection also appeared to enhance the expression of beta-catenin at the cell periphery in transfected epiblast cells (Figure S4). When PTEN siRNA was cotransfected with GFP in a standard transfected-graft experiment, this had little effect on the escape of cells from the transfected primitive-streak graft (Figure S5B). However, it seems likely that this is because PTEN knockdown was not complete, with protein levels falling only slowly over a period of around 20 hr, and that cells escaped from the primitive-streak graft when PTEN expression was still high. Therefore, experiments were performed in which PTEN siRNA was transfected and cell migration was observed in this transfected embryo without grafting (Figure S5D). In this case, the most lateral transfected epiblast cells continue to move toward the streak for many hours after siRNA transfection, before undergoing EMT. In this latter case, a consistent accumulation of cells in the primitive streak was later observed, although this was not seen in control embryos (Figure S5). Furthermore, regression of the node was inhibited, presumably because not enough cells migrate out of the streak, and development was impaired.

The extreme C terminus of PTEN contains a PDZ-domain binding sequence, which is required for the interaction of the phosphatase with several PDZ-domain-containing proteins. Although the role of PDZ-domain-dependent targeting in PTEN function is rather unclear, it is known that the PDZ binding sequence is not required for the general regulation of cellular PtdIns(3,4,5)P_3_ levels and PKB/Akt activity (25, 26 and confirmed during this study [data not shown]). Therefore, we tested a PTEN mutant lacking the last five C-terminal amino acids including the PDZ binding sequence, PTEN ΔPDZ, in the chick-embryo migration assay. Despite its ability to regulate cellular PtdIns(3,4,5)P_3_ levels 26, 27, and despite its retention of catalytic activity in vitro ([26] and Figure S10), PTEN ΔPDZ failed to mediate any detectable effect on the escape of cells from the primitive streak (Figure 3). We noted, however, that although the cells are able to undergo EMT, they displayed aberrant directional migration. This indicates that at least in this assay, the PtdIns(3,4,5)P_3_ phosphatase activity may control the directionality of migration. Expression of the PTEN G129E ΔPDZ mutant did not inhibit EMT, and it allowed normal directional migration of the mesoderm cells (Figure 3F). The fact that PTEN ΔPDZ, which has both lipid and protein phosphatase activity, interferes with directional migration, but expression of PTEN G129E ΔPDZ, which has only a protein phosphatase activity, does not, suggests that it is specifically the lipid phosphatase activity of PTEN that interferes with directional migration when overexpressed.

**Figure 4:**
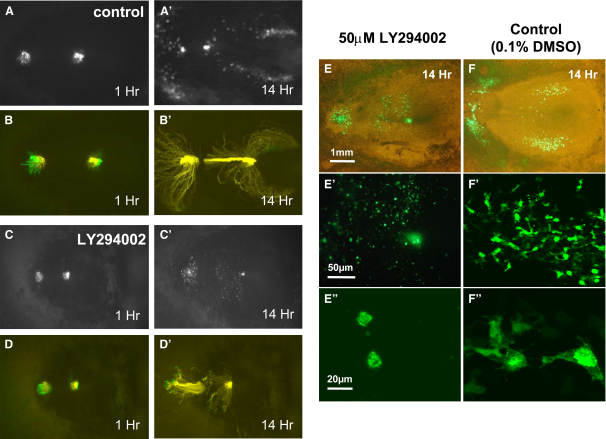
PI3-Kinase Inhibitors Do Not Affect Migration of Cells away from the Primitive Streak, but Interfere with Directional Cell Migration and Cell Polarity (A and C) Fluorescent images of the migration of anterior- and posterior-streak cells in the absence (A) or presence (C) of the PI3K inhibitor LY294002 (50 μM) after 1 and 14 hr of migration. (B and D) The cells tracks of the experiments shown in (A) and (C). (E and F) the morphology of cells after 14 hr of migration in the presence (E) or absence (F) of 50 μM LY294002. Cells in the presence of the inhibitor (E) show a rounded morphology compared to control cells (F), which show a clear polarization in the direction of migration. The scale bars represent 1 mm in (E), 50 μM in (E′), and 20 μm in (E″).

Overexpression of PTEN is expected to result in reduced cellular PtdIns(3,4,5)P_3_ levels, and some of the effects described above, such as the random migration of posterior-streak cells, might be attributed to reduced PtdIns(3,4,5)P_3_ levels. To investigate the effect of low PtdIns(3,4,5)P_3_ levels, we measured the migration of posterior- and middle-streak cells out of the primitive streak in the presence of the phosphoinositide 3-kinase (PI3K) inhibitors LY294002 and PI103 [28]. Both inhibitors showed no detectable effect on the escape of cells from the primitive streak, supporting the above conclusions that EMT is not a PtdIns(3,4,5)P_3_-dependent process. There was, however, a strong effect on directional migration of both middle- and posterior-primitive-streak cells; in the presence of the inhibitors, this directional migration appeared essentially random (Figures 4A–4D and Movie S1). Observation of the migrating cells at higher magnification revealed that cells in the presence of the inhibitor were much more rounded and extended far fewer filopodia and lamellopodia than cells migrating in a control embryo (Figures 4E″ and 4F″). The latter extended many filopodia in the direction of migration, reinforcing the finding that PtdIns(3,4,5)P_3_ has an important role in cell polarization and directional movement.

In order to ensure that the mutations of PTEN used did not have any unexpected effects on protein phosphatase activity, we tested the PTEN mutants C124S, G129E, and ΔPDZ against both the lipid substrate PtdIns(3,4,5)P_3_ and the phosphotyrosine peptide polymer poly-Glu-Tyr(P). These assays showed that deletion of the PDZ binding site did not affect the activity of PTEN in these assays, and they supported the previous data regarding the phosphatase-dead (C124S) and protein-phosphatase-only (G129E) mutants (Figure S10).

### The Role of the C2 Domain and C-Terminal Tail in the Inhibition of Migration

**Figure 5:**
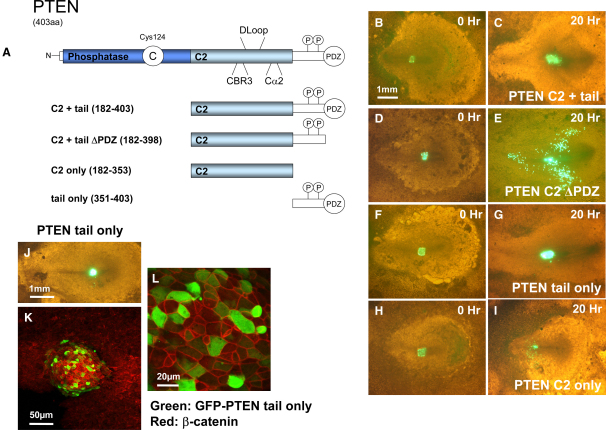
The PTEN C-Terminal Tail Is Sufficient to Block EMT, and the PTEN C2 Domain Is Able to Interfere with the Directional Migration of Cells Escaping the Primitive Streak (A) A schematic diagram of some of the PTEN mutants used in this study is shown. The CBR3 and Cα2 loop mutations comprise the replacement of several exposed basic residues within these loops with alanine residues. (B–I) Embryos were transfected with the mutant GFP-PTEN expression vectors, GFP-PTEN C2 + tail (B and C), GFP-PTEN C2 + tail ΔPDZ (D and E), GFP-PTEN tail only (F and G), and GFP-PTEN C2 only (H and I). Initial (t = 0) and final (t = 20 hr) images are shown of the cell migration assay, merging bright-field and fluorescent images, allowing the outward migration of green fluorescently marked cells to be observed. (J–L) The effect of expression of GFP-PTEN tail only on EMT was investigated as described in Figure 2. Cells of the primitive streak were transfected with vectors encoding GFP-PTEN tail only before grafting into an untransfected host embryo. Development and expression was allowed to proceed for 20 hr before cell migration was assessed by low-power fluorescence microscopy (J), and cellular and tissue morphology were analyzed by immunofluorescence microscopy at medium (K) and high (L) magnification (image sizes as described in Figure 2). The localization of β-catenin is shown in the red channel and GFP-PTEN-tail-only expression in the green channel.

Recent work identified a novel mechanism of action of PTEN in the cell-migration inhibition, mediated by the C2 domain of PTEN [5]. Significantly, for this effect of the C2 domain to be revealed in the full-length protein, the protein phosphatase activity of PTEN was required, apparently to mediate autodephosphorylation of the inhibitory C-terminal phosphorylation sites, particularly Thr383. We tested this effect in the mesoderm migration assay and found that overexpression of a protein containing the C2 domain plus the C-terminal tail of PTEN (aa 182–403) led to a strong inhibition of EMT, similar to that seen through the expression of PTEN G129E (Figures 5B and 5C). This was completely dependent on the expression of the PDZ binding domain because the construct lacking this (C2 + tail ΔPDZ, aa 182–398) failed to inhibit the exit of cells from the primitive streak, but severely impaired the directional migration of the cells (see below).

These data together led to the idea that the observed inhibition of EMT could result from a dominant-negative effect of the PDZ-binding-site-containing tail domain. To test this directly, we studied the expression of the tail domain by itself and found that expression of this domain (aa 353–403) also inhibited EMT completely (Figures 5F, 5G, and 5J–5L). Although the PTEN C124S mutant did not inhibit EMT or cell migration, we found that the lack of inhibition of EMT by the phosphatase-dead PTEN C124S protein could be partially recovered by mutation of the C-terminal phosphorylation sites (Table S1), which may result in the protein's unfolding and exposure of the C2 domain and C-terminal tail or PDZ binding site as previously proposed 5, 29. These data together support the proposal that autodephosphorylation of PTEN is required in order for the C terminus to inhibit migration [5]. However, we find no evidence for the specific significance of Thr383, and our data indicate a novel dominant effect of the C-terminal tail on EMT, in addition to effects of the C2 domain on directional migration as identified in the migration of glioblastoma cells [5]. The directional-impairment effect caused by the expression of PTEN C2 + tail ΔPDZ in the chick embryo was shared by the naked PTEN C2 domain and the C2 domains from both the PTEN-related protein TPTE (see below) and *Dictyostelium* PTEN (Figure 5 and Figure S7). The inhibition of directional migration strongly resembled that seen in the presence of the PI3K inhibitors LY294002 and PI103.

Given that the PTEN C2 domain lacks recognized catalytic or protein interaction motifs, it is not clear how alone it would act to inhibit cell migration. The most likely regions of the domain to be effector motifs would seem to be the extended loops, the long unstructured D loop, and the polybasic CBR3 and Cα2 loops, which have been shown to play a role in membrane interaction and orientation. Indeed, all of these loops play a role in the directional-migration inhibition caused by the C2 domain of PTEN (see Table S1).

To characterize further the inhibition of EMT and directional migration seen with these C-terminal polypeptides, we investigated their effects on PI3K-dependent signaling, finding that expression of the PTEN C2 domain was also found to cause a small but reproducible activation of Akt/PKB in cultured cells lacking PTEN (Figure S8). We also looked at the cellular localization of some of the PTEN constructs used in this study and found that the full-length C2 domain and tail showed a very strong membrane localization, which was strictly dependent on the PDZ binding sequence (Figure S9). The C2 and tail construct lacking this PDZ sequence did not show any significant membrane localization and also lacked all inhibitory activity on EMT. The PTEN-tail-only domain did not show a clear membrane localization; instead, it even showed some nuclear enrichment. The naked C2 domain also does not show a very strong membrane localization. These results suggest that both the C2 domain and the PDZ binding site are necessary for efficient membrane localization.

### Loss of Directional Cell Migration Caused by the PTEN-Related Protein, TPTE, Requires Phosphatase Activity

**Figure 6:**
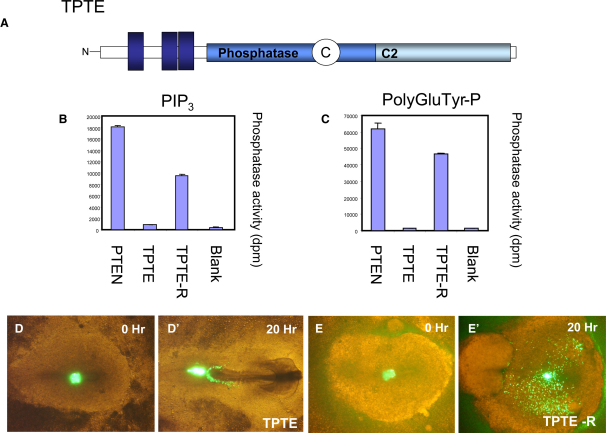
The PTEN-Related Protein TPTE Lacks Phosphatase Activity, but When Reactivated by Mutation Causes Aberrant Directional Migration (A) The TPTE protein-domain structure is shown, containing three N-terminal transmembrane domains, a phosphatase, and a C2 domain. (B and C) The phosphatase activity of PTEN, TPTE, and the “reactivated” mutant TPTE-R against the lipid substrate PtdIns(3,4,5)P_3_ and the phosphorylated peptide polymer polyGluTyr-P are shown. Data is shown as the mean labeled phosphate released from duplicate assays and the range of these duplicates in dpm. (D and E) Expression of GFP-TPTE (D) does not affect EMT or directed cell migration away from the streak, and the expression of the catalytically active GFP-TPTE-R results in random migration of cells exiting the streak (E). Merged bright-field and fluorescence images for the start of the experiment (t = 0) and the end (t = 20 hr) of the experiment are shown.

The PTEN-related protein, TPTE, is very similar in sequence to PTEN through the phosphatase and C2 domains, but lacks a PDZ binding motif and an extensively phosphorylated C-terminal tail. This suggested that we might be able to use TPTE to address the mechanism of action of PTEN in cell-migration assays because it is the C-terminal tail that blocks cell migration out of the primitive streak in this assay and may complicate the analysis of the effects of PTEN by mediating phosphorylation-dependent unfolding of the PTEN protein. We found that although PTEN has robust activity against phosphoinositides, a synthetic peptide [poly-Glu-Tyr(P)], and an artificial substrate (pNPP), several preparations of TPTE had no detectable activity against any of these substrates (Figure S9, Figure 6, and [30]). Remarkably, it proved possible to engineer a “reactivated” mutant of TPTE (TPTE-R), in which a threonine and aspartic acid in the phosphatase P loop were changed to residues found in the corresponding positions in the active phosphatases TPIP and PTEN (Figure 6). Recombinant, bacterially expressed TPTE-R had robust activity against both lipid and polypeptide substrates, which when normalized for full-length protein content indicate that the activities of PTEN and TPTE-R are very similar (Figure 6). We were thus able to gain further insight into the mechanism of action of PTEN in cell-migration assays by making use of TPTE and TPTE-R. When expressed in the anterior primitive streak, GFP-TPTE did not interfere with the directional migration of those cells escaping the streak, whereas GFP-TPTE-R caused strong random migration of these cells (Figures 6D and 6E). This indicates that the phosphatase activity of TPTE-R causes aberrant directional migration of these cells, in agreement with data implicating a role for PtdIns(3,4,5)P_3_ in this process.

## Discussion

Signaling through PI3 kinases and PtdIns(3,4,5)P_3_ has well-established and evolutionarily conserved significance in the directional regulation of cell migration, with evidence that PtdIns(3,4,5)P_3_ is concentrated at the leading edge of many migrating cells 31, 32 and the indication that in some experimental systems, the PtdIns(3,4,5)P_3_ phosphatase activity of PTEN is required for the inhibition of migration by PTEN 6, 33. However, it has also been suggested that the dominant effect of PTEN on cell migration appears to act independently of the PTEN enzymatic activity [5].

We have used mutagenesis to address the mechanism of action by which PTEN expression inhibits cell migration in the developing chick embryo. We have found that overexpression of unfolded PTEN constructs containing the C-terminal PDZ binding sequence result in a strong inhibition of EMT. In the case of expression of full-length PTEN, its protein phosphatase activity is required for this inhibitory effect on EMT to be observed, contributing to the evidence that this phosphatase activity is required to expose the PDZ binding domain. We do not yet know how the PTEN-tail PDZ sequence exerts its inhibitory effect, but it seems likely that it acts in a dominant-negative manner by inhibiting binding of endogenous PTEN to sites at the plasma membrane and that PTEN is required for the proper regulation of EMT. An alternative explanation is that the PTEN-tail-only domain inhibits the binding of another PDZ binding protein, required for EMT, although we favor the former hypothesis. Evidence suggests that PTEN may be required at adherens junctions for the dephosphorylation of PtdIns(3,4,5)P_3_ and protein components such as cadherin and or α- and β-catenin in order to control the dissociation of the cadherin/catenin complexes that is necessary for EMT to occur 20, 21, 34, 35. PTEN would control these components' phosphorylation state, which is necessary for the stabilization of cell-cell junctions, and inhibit EMT, thus explaining part of its action as a tumor suppressor. Our results suggest that endogenous PTEN activity needs to be tightly regulated for EMT to occur in a spatially and temporally coordinated manner. Overexpression of PTEN (and possibly PTEN G129E) would enhance the effects of endogenous PTEN to suppress EMT. Displacement of endogenous PTEN by the tail-only domain would also prevent regulation of these processes and potentially prevent EMT. The experiments in which PTEN expression was knocked down by RNAi are consistent with this proposed model.

Interestingly, we found that overexpression of PTEN was very effective in inhibiting EMT in anterior- and middle-primitive-streak cells but that there was very little effect on EMT in posterior-primitive-streak cells. This shows first of all that the inhibition is not due to some nonspecific effect, but it also shows that the control of EMT in the posterior streak must require a different mechanism possibly involving other adhesion molecules or internal adapter molecules.

The experiments in which PI3K was inhibited through the use of PI3K inhibitors LY294002 and PI103 show that high levels of PtdIns(3,4,5)P_3_ are not necessary for EMT to occur. These experiments, however, did suggest that PtdIns(3,4,5)P_3_ is critically involved in the directionality of migration and that this is presumably through an inhibition of the polarization of the cells in response to factors that guide their migration. This is in line with observations made in other systems such as *Dictyostelium*, neutrophils and fibroblasts, where cells polarize in response to cAMP, FMLP, and PDGF, respectively [31]. The experiments with the PTEN ΔPDZ mutant are in line with this observation. This mutant lacks the inhibitory effect on EMT, presumably because of a lack of PDZ-binding-site-mediated targeting to sites required for the regulation of EMT. Therefore, cells expressing this mutant undergo EMT, and we could investigate the effect of the PTEN on cell migration. These experiments show that overexpression of PTEN ΔPDZ results in random cell migration, whereas expression of PTEN G129E ΔPDZ, which lacks the lipid phosphatase activity, does not result in random cell migration. These findings indicate that it is the lipid phosphatase activity of PTEN that interferes with the directionality of cell migration and that the protein phosphatase activity has no significant effect on this property. Random cell migration is also observed in the case of expression of TPTE-R. Despite its plasma-membrane localization, there is no significant effect of TPTE on EMT, possibly because it is not targeted to the correct sites in the membrane to inhibit EMT. The lipid phosphatase activity, however, should effectively modulate cellular PtdIns(3,4,5)P_3_ levels and could result in the inhibition of directional migration. Although there is a good correlation between the lipid phosphatase activity of the PTEN and TPTE constructs used and their capacity to cause aberrant directional migration, it is possible that this activity does not directly block PI3K-dependent polarization and cell-autonomous chemotaxis, but rather interferes with other aspects of the complex migratory phenotype observed in the developing embryo.

The C2 domain of PTEN from a variety of species is able to inhibit directional cell migration in the absence of phosphatase activity, resembling very much the phenotype observed after inhibition of PI3K. The finding that expression of the PTEN C2 domain constructs in cultured cells could activate Akt/PKB suggests that it is possible that the C2 domain of PTEN directly interferes with PI3K-dependent directional sensing through an unknown mechanism, resulting in the observed random migration. Random migration could be an explanation for the effect of the C2 domain observed in the wound-healing assay [5] because this will result in less efficient migration into a scratch-wound area.

Several pieces of evidence indicate that TPTE lacks significant phosphatase activity. TPTE is most similar in sequence to, and evolutionarily appears to have arisen from, the active phosphatases PTEN and TPIP (47% and 92% identity through the phosphatase domain, respectively). In our experiments in which PTEN had good activity against three different substrates, TPTE lacked all detectable activity. Strikingly, however, when TPTE was mutated to have the same P loop sequence as PTEN and TPIP, this mutant protein had strong phosphatase activity against all three substrates. This strongly suggests that these changes have occurred in the TPTE sequence during evolution to produce a protein lacking phosphatase activity, although the reason for this is unclear.

In conclusion, it appears that PTEN may play a role in two distinct processes controlling cell migration during embryonic development. It seems to be critical for the control of EMT. This action appears to require its protein phosphatase activity, via both autodephosphorylation to expose the tail domain and possibly also protein phosphatase activity against other protein substrates. Furthermore, through its lipid phosphatase activity, it appears to be able to control cell polarization and directionality of mesodermal cell migration through the regulation of cellular PtdIns(3,4,5)P_3_ levels. These two actions may also be important in the development of tumors that mostly arise in epithelia and then undergo EMT before metastasis.

## Experimental Procedures

### Embryo Manipulation and Cell GFP Labeling

Brown Leghorn chick embryos (Henry Stewart, Lincolnshire) were incubated at 37°C in a tray-rocking incubator until they reached HH 2–3 (Hamburger and Hamilton stage 2–3) [36]. New cultures [37] and early chick (EC) cultures [38] were prepared, and transfection was achieved by electroporation of 0.5 μl plasmid DNA at a concentration of 1.0 mg/ml, microinjected into the space between the vitellin membrane and the epiblast, next to the anterior primitive streak, in HH2-3 embryos, by using a microinjector (FemtoJet, Eppendorf). Embryos were electroporated by applications of two successive 50 ms square pulses of 10 V through two parallel electrodes, 1.5 mm apart, by using a custom-built electroporator (Isolated Stimulator Model DS2, Digitimer, United Kingdom). After electroporation, the embryos were further incubated at 38°C for 3–5 hr, after which well-labeled GFP-positive primitive-streak tissue from anterior, middle, or posterior region of the streak was grafted into a host embryo of the same stage as the donor, from which an equivalent piece of primitive streak was removed by using a tungsten needle. The embryos were incubated at 38°C for 1 hr, after which they were photographed and time-lapse imaging was started. For one-sided electroporation, the polarity of the pulses was kept constant, whereas for electroporation of both sides of the embryo, the polarity of the electrodes was switched between pulses.

### Time-Lapse Imaging

Imaging of cell movement during early gastrulation was performed as described previously [19]. Labeled embryos were incubated in a custom-built microscope chamber, kept at 38°C with water-saturated heated air (AIR-THERMZ, serial: 54833-L048) and mounted on a Zeiss Axiovert 100 inverted microscope with a plan-NEOFLUAR 2.5×/0.075 objective (ZEISS) and Hamamatsu Orca-ER camera [39]. Images were collected with Simple PCI software. Both bright-field and fluorescence images were taken every 3 min. Cell-movement tracks were generated by successive logical addition of images with macros written with the Optimas VI imaging library. In a typical experiment, we prepared two successful grafts starting with around ten embryos; one embryo was chosen for filming, and the remaining embryos were photographed at the beginning and end of the experiment. Each experiment was repeated at least three or four times (see Table S1).

### Antibodies, Western Blotting, and Immunocytochemistry

Cell culture, lysis, western-blotting procedures, and assays of cellular Akt/PKB activity were as previously described [40]. DF1 chicken fibroblasts were kindly provided by Cheryl Tickle (University of Dundee). Antibodies against PTEN were purchased from Cascade Bioscience (6H2.1 monoclonal, used for all studies of endogenous cPTEN expression) and Santa Cruz (A2B1 monoclonal), and those against PKB/Akt and E-Cadherin (L-CAM) were purchased from Cell Signalling Technologies and the Developmental Studies Hybridoma Bank (University of Iowa), respectively. Antibodies against β-actin were from Sigma, and those raised against Glutathione S-Transferase and GFP were kindly provided by James Hastie and Hilary McLaughlin (Division of Signal Transduction Therapy, Dundee University). For immunocytochemistry, chick embryos were fixed overnight at 4°C in 4% paraformadelhyde in PBS (pH 7.4) and washed three times in PBS, followed by inactivation of endogenous peroxidase by incubation with 0.3% H_2_O_2_ in PBS for 30 minutes. Embryos were washed three times in PBS followed by blocking in PBT (2% Bovine Serum Albumin, 1% Triton-X; 1% Tween 20 in PBS) for 1 hr at room temperature. The embryos were incubated in anti-PTEN (6H2.1) at 1:100 in PBT overnight at 4°C, followed by a further overnight incubation in peroxidase-conjugated anti-mouse (Promega) 1:1000 dilution. Anti-β-catenin (Sigma clone 15B8) was used in a 1:100 dilution in PBT, followed by peroxidase anti-mouse (1:1000 dilution). Detection was performed by using the tyramide-signal-amplification system (Molecular Probes) with Alexa Fluor 555, according to the manufacturer's instructions.

## Supplemental Data

Document S1. Experimental Procedures, Ten Figures, and One Table

Movie S1. Migration of Anterior- and Posterior-Streak Cells Expressing GFP, Wild-Type GFP PTEN, or GFP in the Presence of the PI3-Kinase Inhibitor LY294002 LY294002 was used at 50 μM. The sequences were recorded over 7 hr of development. The panels on the left show the migration of the individual cells, and the panels on the right show the traces of the cells during this period of migration.
