# Supplementary material for: PtdIns(3,4,5)P3-Dependent and -Independent Roles for PTEN in the Control of Cell Migration
Source: Curr Biol. 2007 Jan 23;17(2):115–25. doi: 10.1016/j.cub.2006.12.026 (PMC1885949; doi:10.1016/j.cub.2006.12.026)
Supplement: Document S1. Experimental Procedures, Ten Figures, and One Table [file mmc1.pdf]

Supplemental Data

PtdIns(3,4,5)P_3-Dependent and -Independent Roles for PTEN in the Control of Cell Migration

Nick R. Leslie, Xuesong Yang, C. Peter Downes, and Cornelis J. Weijer

Supplemental Experimental Procedures

Expression-Vector Construction

Oligonucleotide sequences used for the cloning and mutagenesis in this study are referred to here and detailed below. Expression vectors based upon EGFP-C2 (Clontech) for wild-type GFP-PTEN (human) and the mutants C124S, G129E, M-CBR3, and 399stop (here called ΔPDZ) have been previously described [S1, S2]. Site-directed mutagenesis of GFP-PTEN was performed with the oligonucleotide PTEN Cα2S and its reverse complement in order to make to make GFP-PTEN Cα2 (see below). GFP-PTEN ΔDLoop was produced by deletional PCR of GFP-PTEN with primers PTEN DLoop S and PTEN DLoop AS.

Expression vectors for the C2 domain of PTEN were produced by PCR from full-length or 399stop mutant PTEN cDNAs with the primers PTEN C2 5′ BsrGI with either PTEN C2short 3′ NotI or PTEN C2 3′ NotI. Expression vectors for the PTEN tail only were

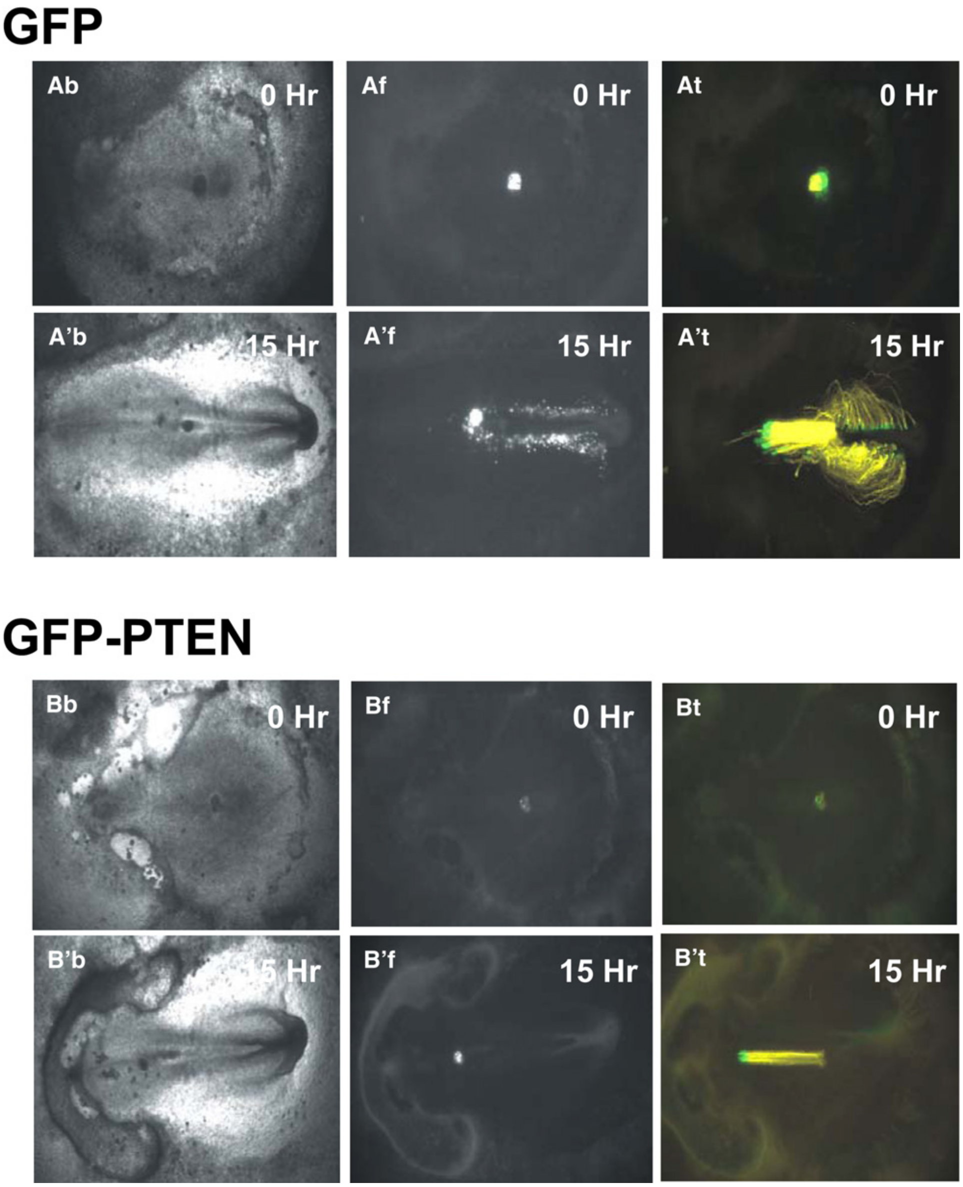

Figure S1. Migration of Anterior-Streak Cells Expressing either GFP or a GFP-PTEN Fusion Protein

([A], top set of six panels) shows cells expressing GFP, and ([B], lower set of six panels) shows cells expressing a GFP-PTEN fusion protein. Data in each column show, from left to right, bright-field (b), fluorescence (f), and cell-track (t) images. The rows in each set indicate times (0 and 15 hr) after grafting. Note that cells expressing GFP move out and forward, whereas cells expressing GFP-PTEN do not move out of the anterior streak, but the streak itself continues to regress.

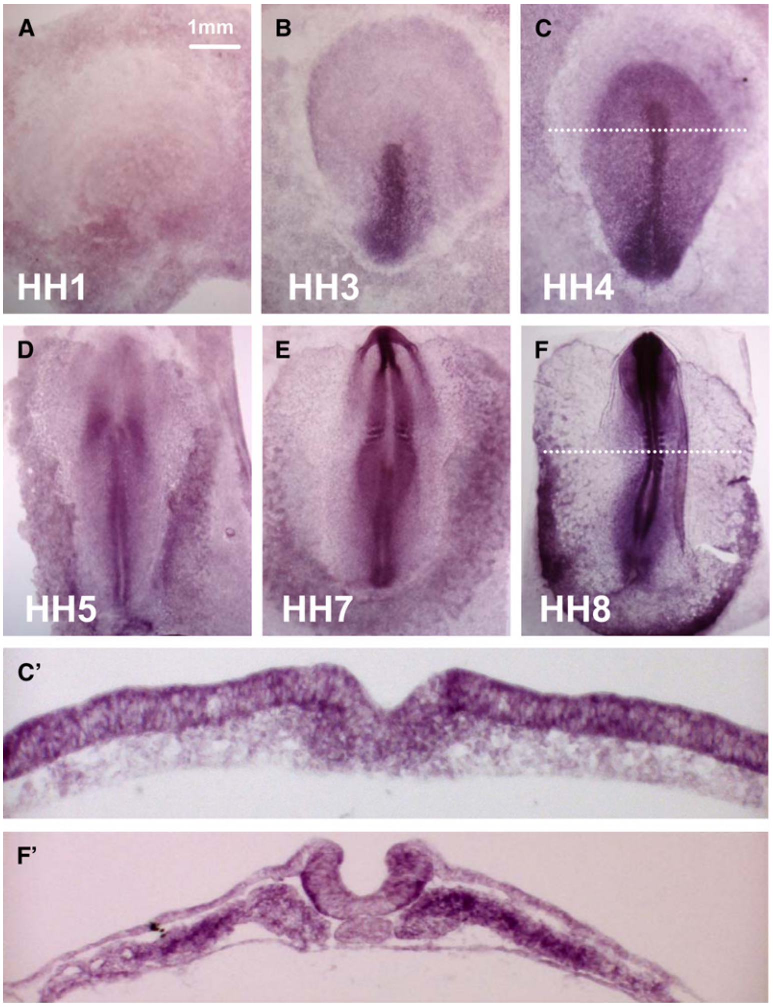

Figure S2. PTEN Expression in Chick Embryos at Developmental Stages HH1–HH8

Expression of PTEN mRNA was addressed by in situ hybridization in the developing chick embryo. Sections taken as indicated by the dashed line are shown in (C′) and (F′).

similarly generated by PCR with the sense primer PTEN351tail Eco-S. A vector expressing the C2 domain of TPTE was produced similarly by using the TPTE C2 clone S and TPTE C2 clone AS.

Construction of an expression vector for GFP-TPTE based upon EGFP-C1 has been previously described [S3]. All experiments here use expression of TPTEγ from the nomenclature of Tapparel et al. [S4]. TPTE-R was produced by using the primer TPTE reactivate S and its reverse complement. Mutagenesis of the potential TPTE active-site cysteine residue was performed, but caused the protein to express poorly in *E. coli*, in the chick embryo, and in cultured HEK293 cells, presumably through effects on protein stability.

Mutagenesis of the C-terminal phosphorylation sites was performed with previously described oligonucleotide sequences [S5] S380A, T382A, T383A, S385A, A3 (triple mutant S380A, T382A, T383A), and D3 (triple mutant S380D, T382D, T383D).

Oligonucleotides

In each case, the indicated oligonucleotide sequence and its reverse complement were used for PCR mutagenesis.

Oligonucleotides for site-directed mutagenesis were as follows: PTEN Ca2 S, 5′-CAAAAAATGATCTTGACGCAGCCGGAGCAGACGCTGCAAACGCATACTTTTCTCC-3′ and TPTE reactivate S 5′-CACTGTAAAGGAGGCAAGGGTAGAACAGGAACTATGG-3′.

Oligonulceotides for deletion mutagenesis were as follows: PTEN DLoop S, 5′-AATGACAAGGAATATCTAGTACTTAC-3′ and PTEN DLoop AS, 5′-TTCCTCTGGTCCTGGTATGAAG-3′.

Oligonucleotides for subcloning of C2 domain and tail fragments were as follows: PTEN C2 5′ BsrGI, 5′-GGTGTACAAGACCGAGCTGCAGTTAAAGAATCATCTGG-3′; PTEN C2 3′ NotI, 5′-GGGCGGCCGCTCAGACTTTTGTAAT-3′; PTEN C2short 3′ NotI, 5′-GGGCGGCCGCTCACGGCTCCTCTACTG-3′; PTEN351tail Eco-S, 5′-GGCGAATTCGTAGAGGAGCCGTCAAATCC-3′; TPTE C2 clone S, 5′-TACAGAATTCAAACATCTCTACAACTGG-3′; and TPTE C2 clone AS, 5′-CATTGTCGACTGATCACATTTTCTCGCCAAAAAG-3′.

Protein Expression and Purification

Glutathione S-transferase fusion proteins were expressed from previously described vectors based upon pGEX6P1 in *E. coli* BL21 cells induced overnight at 20°C with 100 μM IPTG [S3]. Expressed protein was purified with Glutathione sepharose 4B (both Amersham Pharmacia Biotech) following standard procedures and manufacturer's guidelines. Proteins were stored at −80°C in elution buffer (50 mM Tris [pH 7.4], 150 mM NaCl, 20 mM reduced glutathione, 1 mM EGTA, 0.01% [vol/vol] Brij-35, 0.1% [vol/vol] 2-mercaptoethanol, 10 μg/ml leupeptin, 100 μM PMSF, and 1 mM benzamidine) with 15% (vol/vol) added glycerol.

RNA-Interference Experiments

An siRNA "smartpool" targeting the chicken PTEN gene was purchased from Dharmacon. siRNA was diluted to a concentration of 1 μM in 20 mM KCl, 6 mM Hepes (pH 7.5), and 200 μM $MgCl_2$ and electroporated according to the protocol and conditions of the plasmid constructs. Standard whole-mount in situ hybridization used an antisense probe against cPTEN according to the method of Wilkinson and Nieto [S6].

Substrate Preparation and Phosphatase Assays

Poly-Glu:Tyr (4:1) (Sigma) was phosphorylated by using 1 mg of polymer in 0.2 ml of a kinase buffer (50 mM Hepes [pH 7.4], 12 mM $MgCl_2$, 1 mM EGTA, 1 mM 2-mercaptoethanol, and 18.5 MBq

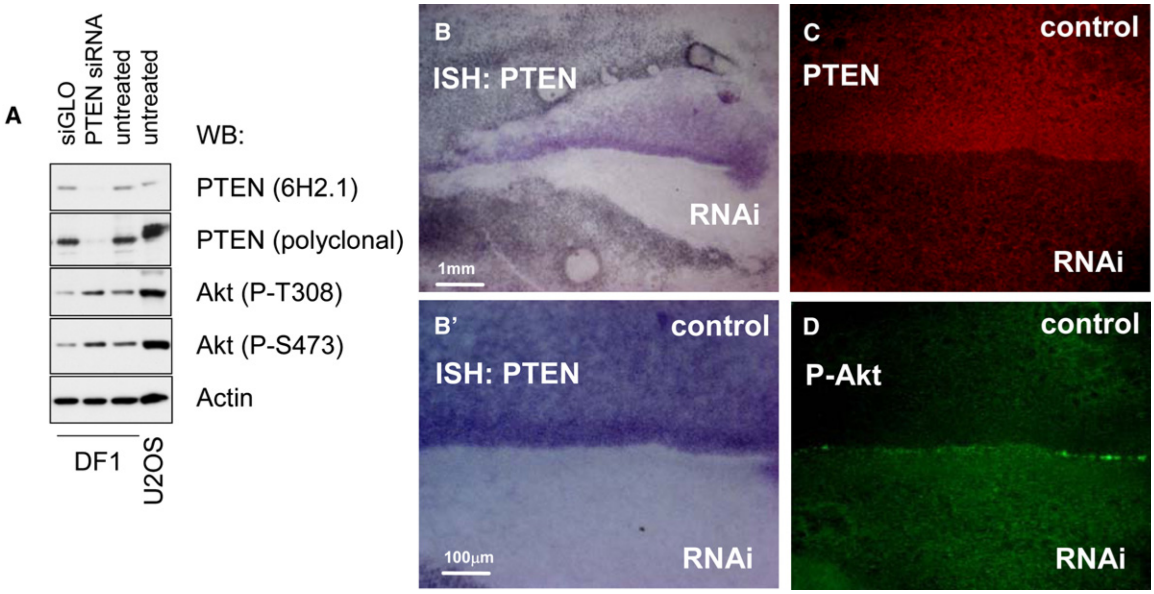

Figure S3. Knockdown of PTEN by RNAi Increases Akt/PKB Phosphorylation

(A) Chicken DF1 fibroblasts were transfected with either control fluorescently labeled siRNA (siGLO) or siRNA targeting the chicken PTEN gene, or they were left untransfected. After 24 hr, cells were lysed and PTEN and actin expression and the phosphorylation of Akt/PKB were investigated by western blotting. A sample of human U2OS cell lysate was also included to assess the recognition of chicken proteins by the antibodies used.

(B–D) HH2 embryos were electroporated on one side only with PTEN siRNA PTEN (the lower side as seen above). The localized effects on the expression of PTEN mRNA and protein were observed 24 hr later by in situ hybridization (ISH, [B] and [B′]) and immunofluorescence, respectively (C), and on Akt phosphorylation with antibodies specific for phospho-Ser473 Akt (D).

γ-[^{33}P] ATP), with incubation with 1 μg insulin receptor kinase domain (Upstate) for 1 hr at 32°C before addition of 0.5 μg further kinase and incubation for another hour. The reaction was stopped by addition of 0.5 ml 20% TCA at 4°C and centrifuged at 14,000 × g for 10 min, and the pellet washed once in fresh 10% TCA. The pellet was then resuspended in 500 μl 1M Tris (pH 7.4) and dialysed twice at 4°C against 10 mM Tris (pH 7.4) by using a 3,500 MW cut-off dialysis cassette (Pierce). 3-[^{33}P] PtdIns(3,4,5)P_3 was prepared as previously described [S7].

PtdIns(3,4,5)P_3 assays were conducted with substrate vesicles prepared by sonication of 100 μM phosphatidylcholine, 10 μM unlabeled PtdIns(3,4,5)P_3, and 100,000 dpm 3-[^{33}P] PtdIns(3,4,5)P_3. These were incubated in 50 mM Tris [pH 7.4], 150 mM NaCl, 1 mM EGTA, and 10 mM DTT with 1 μg enzyme for 30 min at 32°C. PolyGlu-Tyr(P) phosphatase assays were conducted in 50 mM Tris (pH 7.4), 1 mM EGTA, and 10 mM DTT with 1 μg of enzyme and 100,000 dpm (approximately 1 μg) of phosphorylated substrate per assay, also at 32°C for 30 min. Reactions were terminated directly by the addition of 500 μl of ice-cold 1M perchloric acid and 100 μg/ml BSA, left on ice for 30 min, and spun at 15,000 × g at 4°C for 10 min. The supernatant was removed, and ammonium molybdate was added to a final concentration of 10 mg/ml. After extraction with 2 vol of toluene/isobutanol (1:1 vol/vol), the upper phase was removed and radioactivity was determined by scintillation counting. pNPP assays were performed with 2 μg of enzyme, 20 mM pNPP (Sigma), 50 mM Tris (pH 7.4), 1 mM EGTA, and 10 mM DTT, and activity was measured by absorbance at 405 nm.

Supplemental References

S1. Leslie, N.R., Bennett, D., Gray, A., Pass, I., Hoang-Xuan, K., and Downes, C.P. (2001). Targeting mutants of PTEN reveal distinct subsets of tumour suppressor functions. Biochem. J. *357*, 427–435.

S2. Leslie, N.R., Gray, A., Pass, I., Orchiston, E.A., and Downes, C.P. (2000). Analysis of the cellular functions of PTEN using catalytic domain and C- terminal mutations: Differential effects of C-terminal deletion on signalling pathways downstream of phosphoinositide 3-kinase. Biochem. J. *346*, 827–833.

S3. Walker, S.M., Downes, C.P., and Leslie, N.R. (2001). TPIP: A novel phosphoinositide 3-phosphatase. Biochem. J. *360*, 277–283.

S4. Tapparel, C., Reymond, A., Girardet, C., Guillou, L., Lyle, R., Lamon, C., Hutter, P., and Antonarakis, S.E. (2003). The TPTE gene family: Cellular expression, subcellular localization and alternative splicing. Gene *323*, 189–199.

S5. Vazquez, F., Ramaswamy, S., Nakamura, N., and Sellers, W.R. (2000). Phosphorylation of the PTEN tail regulates protein stability and function. Mol. Cell. Biol. *20*, 5010–5018.

S6. Wilkinson, D.G., and Nieto, M.A. (1993). Detection of messenger RNA by in situ hybridization to tissue sections and whole mounts. Methods Enzymol. *225*, 361–373.

S7. McConnachie, G., Pass, I., Walker, S.M., and Downes, C.P. (2003). Interfacial kinetic analysis of the tumour suppressor phosphatase, PTEN: Evidence for activation by anionic phospholipids. Biochem. J. *371*, 947–955.

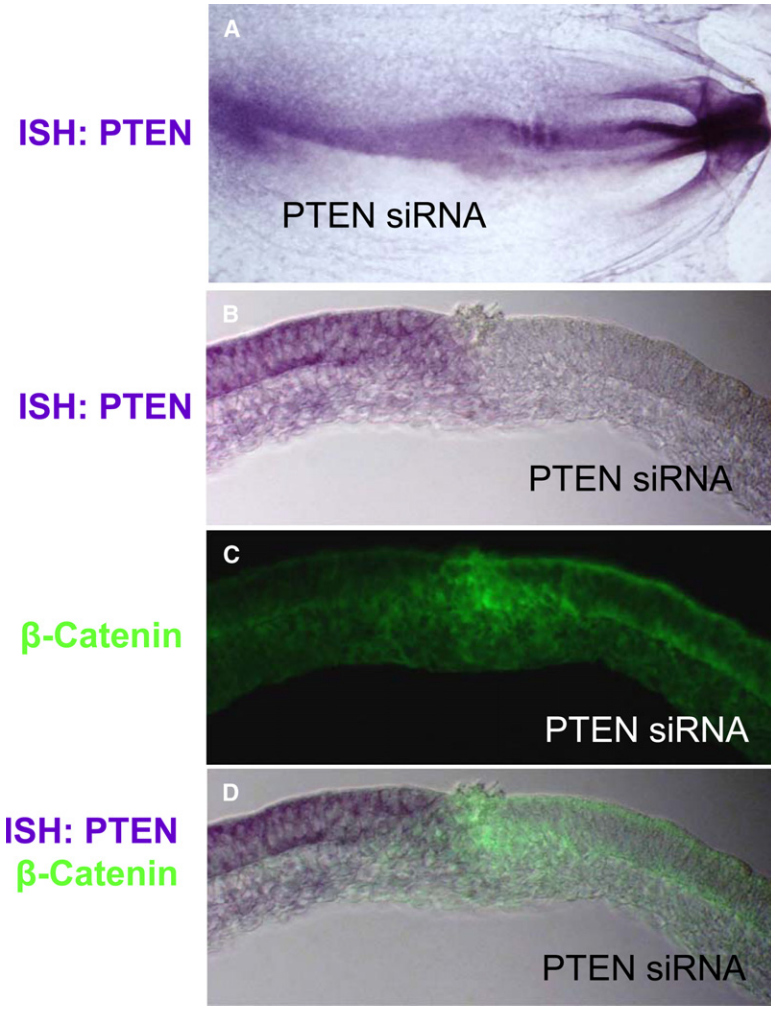

Figure S4. PTEN siRNA Transfection Increases Expression of β-Catenin

HH2 embryos were transfected with PTEN siRNA on one side only. In (A), this corresponds to the lower half of the embryo, and in the embryo cross-sections (B–D), this corresponds to the right-hand side of the image. After 20 hr, expression of PTEN mRNA was assessed by in situ hybridization (blue stain in [A], [B], and [D]), and β-catenin expression was assessed by antibody staining (green fluorescence in [C] and [D]).

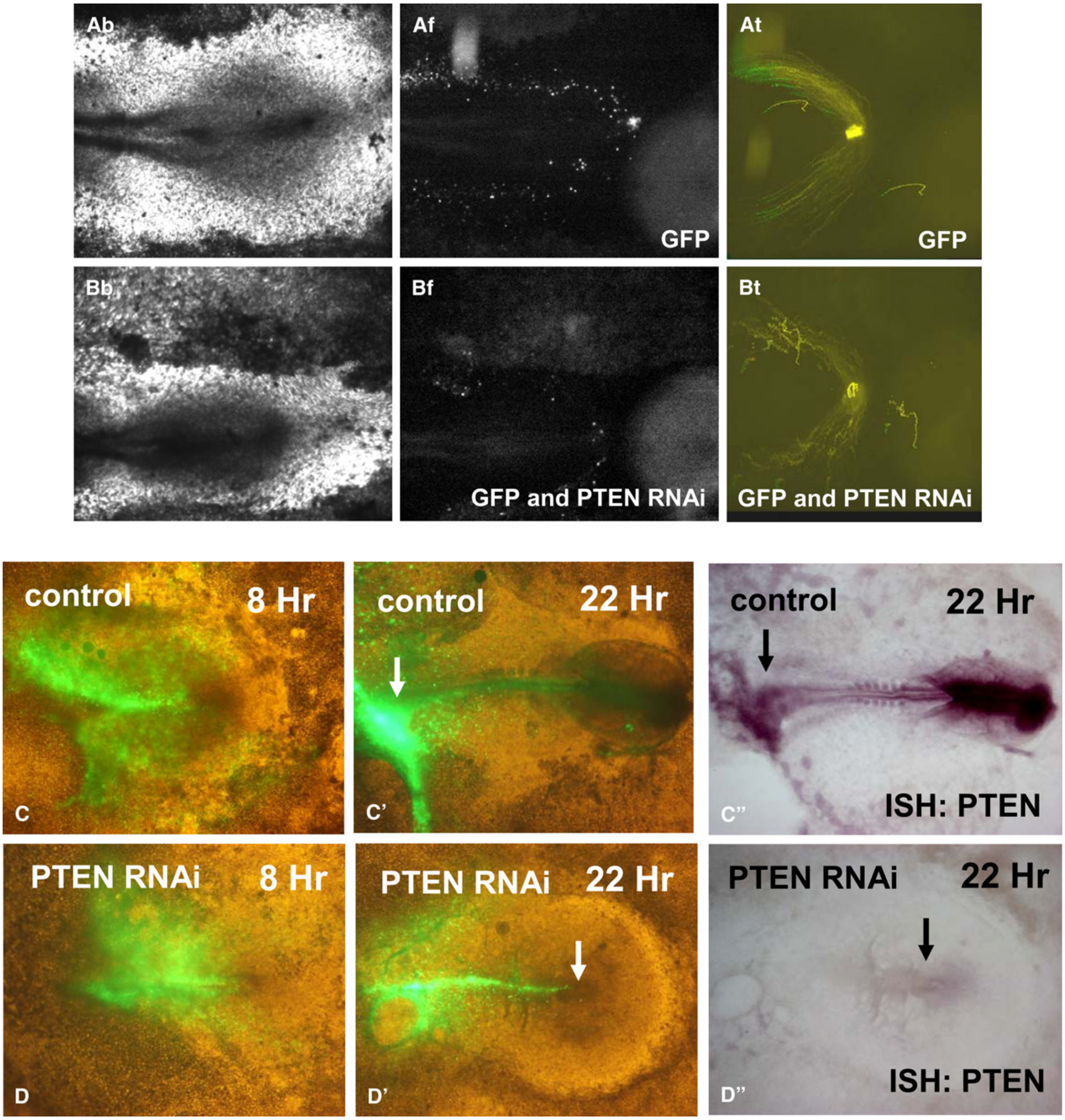

Figure S5. Effects of PTEN RNAi on Cell Migration

Cells were either transfected with GFP (A and C) or cotransfected with GFP and PTEN siRNA (B and D).

(A and B) Eight hours after transfection, cells from the anterior primitive streak were grafted into an untransfected host embryo, and the movement of the cells was recorded for 15 hr. The three data panels show, from left to right, bright-field (b), fluorescence (f), and cell-track (t) images.

(C and D) Embryos transfected with either GFP (C) or PTEN siRNA and GFP (D) and in which cell migration is observed without grafting. Observation started 8 hr after transfection. Merged fluorescence-bright-field images are shown from the beginning and end of the observation period (8 hr [C and D] and 22 hr [C′ and D′]) to show the final migration pattern of the transfected cells through the embryo. Arrows indicate the position of Henson's node, illustrating the failure of primitive-streak regression in PTEN-siRNA-transfected embryos. At the end of the experiment, most of the cells located at the midline of the control embryo are those that have migrated out from the streak to form axial mesoderm, somites, and lateral-plate mesoderm, whereas many of the cells in the RNAi embryos have failed to migrate away and can be seen as highly fluorescent clusters, remaining in the streak. A cross-section of this streak in embryos transfected with PTEN siRNA shows the accumulation of epiblast cells in the epidermal layer, consistent with a failure to undergo EMT (data not shown). In situ hybridization (ISH) for PTEN was performed to verify the knockdown of PTEN expression (C″ and D″).

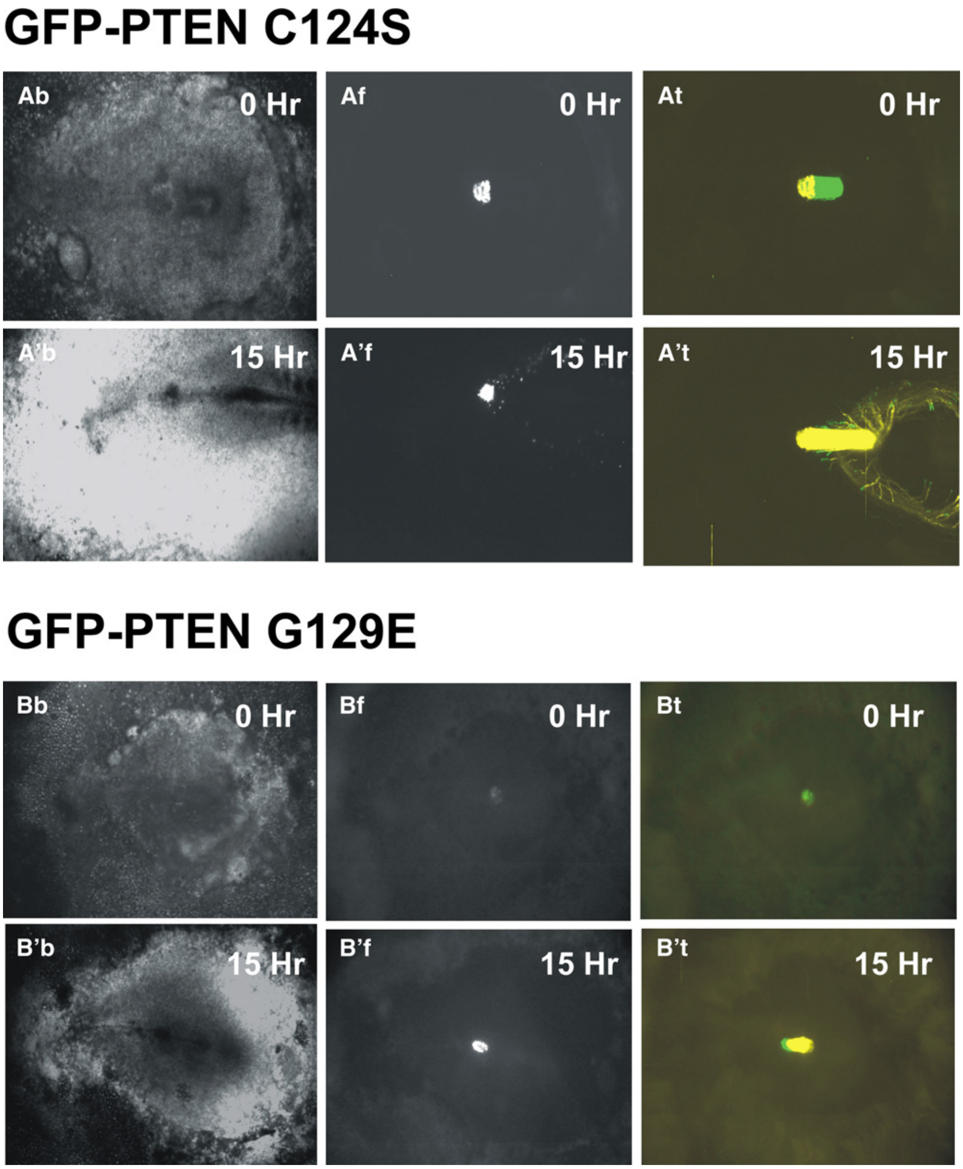

Figure S6. Migration of Anterior-Streak Cells Expressing either Phosphatase-Dead GFP-PTEN C124S or a Protein-Phosphatase-Only GFP-PTEN G129E Fusion Protein

([A], top set of 6 panels) shows cells expressing phosphatase-dead GFP-PTEN C124S, and ([B], lower set of six panels) shows cells expressing a protein-phosphatase-only GFP-PTEN G129E fusion protein. Data in each column show, from left to right, bright-field (b), fluorescence (f), and cell-track (t) images. The rows in each set indicate times (0 and 15 hr) after grafting. Note that cells expressing PTEN C124S move out and forward, whereas cells expressing PTEN G129E do not move out of the anterior streak.

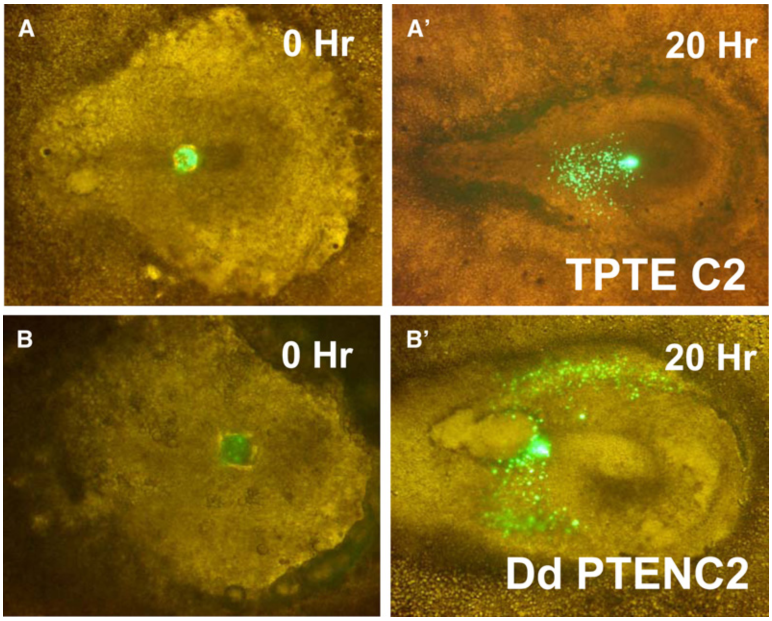

Figure S7. Expression of the C2 Domain of TPTE or of *Dictyostelium* PTEN Interferes with Directional Cell Migration

Embryos were transfected with expression vectors encoding (A) GFP TPTE C2 or (B) GFP DdPTEN C2 and observed during development as described in the text. Merged bright-field and fluorescence images for the start of the experiment (t = 0) and the end (t = 20 hr) of the experiment are shown. Expression of each C2 domain interferes with the normal directional patterns of migration through the embryo; see, for example, Figure 1 and Figure S1.

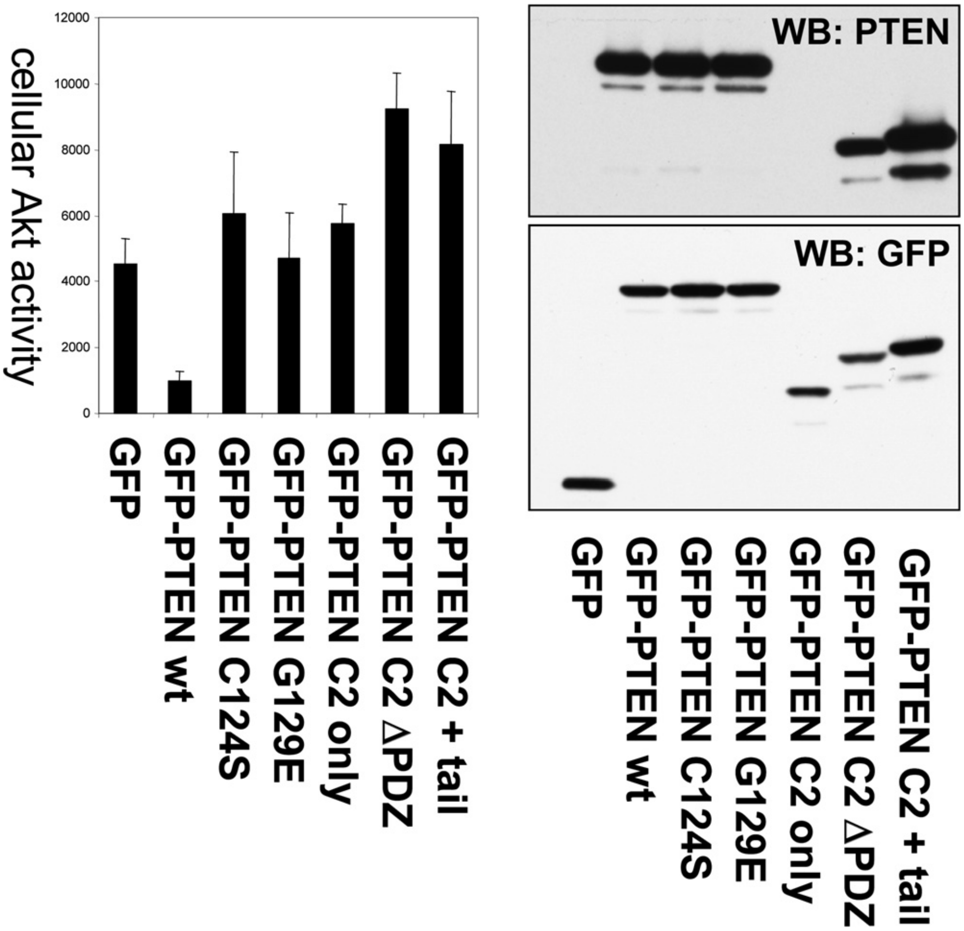

Figure S8. Overexpression of the PTEN C2 Domain Activates Akt/PKB

PTEN null 1321N1 astrocytoma cells were infected with baculoviruses encoding GFP or the indicated GFP-PTEN fusion proteins. Cells were incubated for 24 hr and lysed before the endogenous Akt/PKB was immunoprecipitated and assayed in vitro against the peptide substrate Crosstide. Activity data are presented as the mean + standard deviation. Expression of GFP and PTEN in these lysates was assessed by western blotting with antibodies against GFP and PTEN. The A2B1 monoclonal antibody used to detect PTEN recognizes an epitope that is in the C-terminal tail and is absent in GFP-PTEN C2 only.

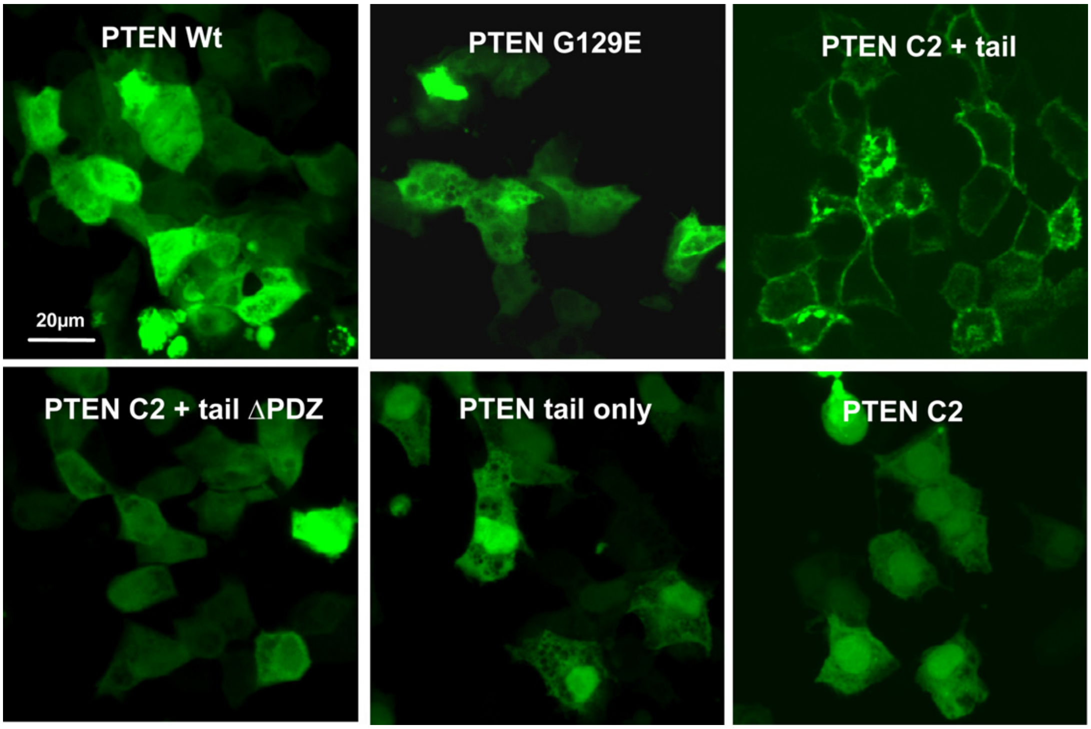

Figure S9. The Localization of the Indicated GFP-PTEN Fusion Proteins Is Shown

Embryos were transfected and development was allowed to proceed for 15 hr before cells were photographed. Only the GFP-PTEN C2 + tail construct seems strongly membrane localized, with the C2-only and C2 ∆PDZ constructs displaying some weak membrane enrichment.

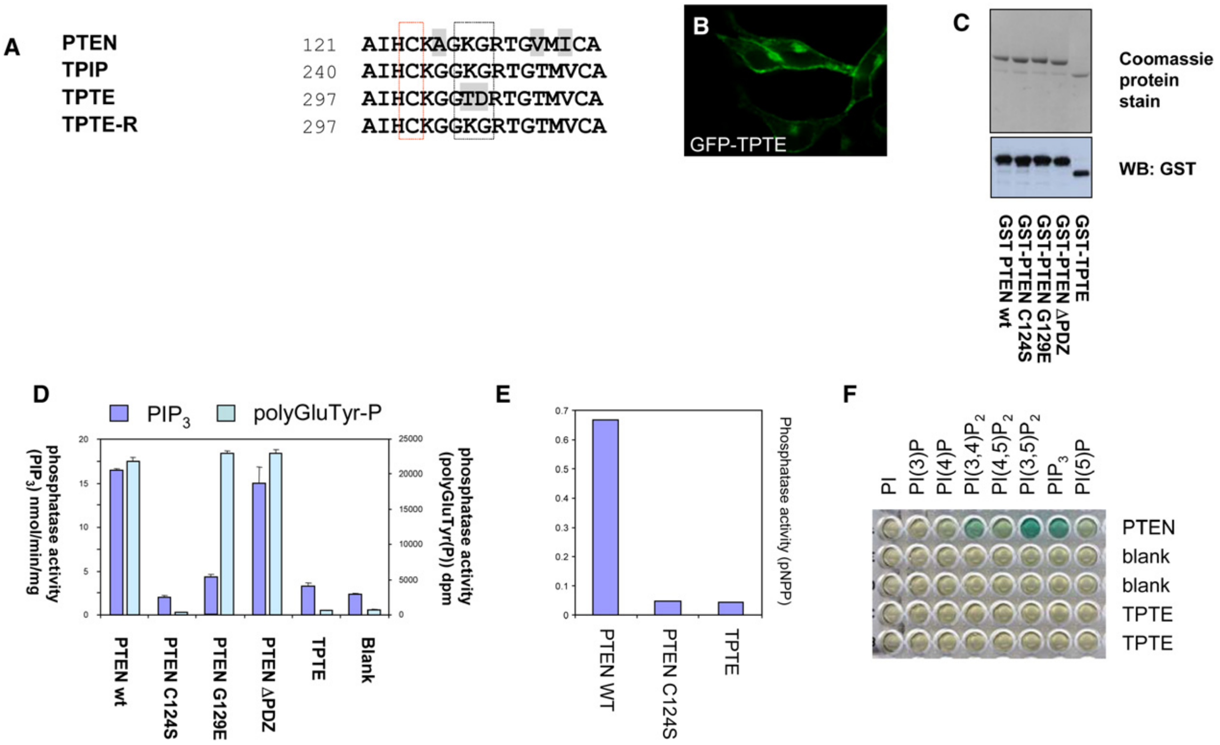

Figure S10. TPTE Lacks Detectable Phosphatase Activity

(A) An alignment of the active-site P loop of PTEN, TPIP, and TPTE is shown, with the PTEN catalytic-cysteine residue boxed in red. The black boxing shows the threonine and aspartic-acid residues at which TPTE differs from the active phosphatases PTEN and TPIP in this region and the mutation of these residues performed in reactivated TPTE (called TPTE-R).

(B) When expressed as a GFP-fusion protein in HEK293 cells, TPTE is located largely on the plasma membrane.

(C) The protein preparations used in the presented phosphatase assays (here and in Figure 6) are shown with Coomassie total-protein stain and anti-GST western blotting (WB). The GST-TPTE fusion protein contains all of the main intracellular regions of the protein, lacking the N-terminal transmembrane domains.

(D) The activity of the indicated PTEN and TPTE proteins was assayed with either ^{33}P-radiolabeled PtdIns(3,4,5)P_3 or phosphorylated polyGluTyr as substrate. Data are shown as mean activity and standard deviation from triplicate assays.

(E) Activity is shown against the artificial substrate para-nitrophenol phosphate (pNPP) as the mean measured optical density (OD) from duplicate samples.

(F) TPTE lacks activity against other phosphoinositide substrates. Two micrograms of recombinant GST-PTEN or GST-TPTE were incubated with each phosphoinositide lipid for 1 hr at 30°C before released inorganic phosphate was measured with malachite green reagent. Lipids were present as synthetic diC8-soluble compounds at 100 μM. An image of the experiment is shown. No significant increase in measured OD at 620 nM was detected in TPTE samples relative to control (data not shown).

Table S1. A Summary of the Data from Chick-Embryo Migration Assay Using PTEN Mutant Proteins

Effect	PTEN wt	C124S (phosphatase dead)	G129E (protein phosphatase only)	C2 only (182–353)	C2 + tail (182–403)
WT					
EMT block	8/9	0/9	9/10	1/6	7/9
Cell migration: directed	1/9	8/9	1/10	0/6	2/9
Cell migration: random	0/9	1/9	0/10	5/6	0/9
ΔPDZ					
EMT block	0/8		1/6		1/7
Cell migration: directed	1/8		5/6		0/7
Cell migration: random	7/8		0/6		6/7
CBR3 Ala					
EMT block	2/4		4/4	1/7	
Cell migration: directed	0/4		0/4	2/7	
Cell migration: random	2/4		0/4	4/7	
Cα2 Ala					
EMT block	4/4		2/7	1/5	
Cell migration: directed	0/4		5/7	4/5	
Cell migration: random	0/4		0/7	0/5	
Δ D Loop					
EMT block				4/12	
Cell migration: directed				4/12	
Cell migration: random				4/12	
S380A					
EMT block		4/6			
Cell migration: directed		2/6			
Cell migration: random		0/6			
T382A					
EMT block		3/4			
Cell migration: directed		1/4			
Cell migration: random		0/4			
T383A					
EMT block		3/6			
Cell migration: directed		2/6			
Cell migration: random		1/6			
S385A					
EMT block		2/4			
Cell migration: directed		2/4			
Cell migration: random		0/4			
A3 (380/382/383)					
EMT block	3/4	4/8	4/7		4/8
Cell migration: directed	0/4	3/8	2/7		4/8
Cell migration: random	1/4	1/8	1/7		0/8
D3 (380/382/383)					
EMT block	3/5				
Cell migration: directed	1/5				
Cell migration: random	1/5				

PTEN mutant and double-mutant proteins are identified from the combinations of the top row and the left column. Each experimental set shows the three possible experimental outcomes: an EMT block with cells failing to escape the primitive-streak graft; random cell migration, with cells escaping the graft, but migrating randomly through the embryo; or directed cell migration, with cells escaping from the graft and following their normal migratory tracks through the embryo. Numbers represent the frequency of each outcome and the number of experiments performed.
